# MYC overexpression with its prognostic and clinicopathological significance in breast cancer

**DOI:** 10.18632/oncotarget.21501

**Published:** 2017-10-05

**Authors:** Jingkun Qu, Xixi Zhao, Jizhao Wang, Xu Liu, Yan Yan, Lin Liu, Hui Cai, Hangying Qu, Ning Lu, Yuchen Sun, Feidi Wang, Jiansheng Wang, Jia Zhang

**Affiliations:** ^1^ The Second Department of Thoracic Surgery, The First Affiliated Hospital of Xi’an Jiaotong University, Xi’an, Shaanxi 710061, P.R. China; ^2^ Department of Oncology, The Second Affiliated Hospital of Xi’an Jiaotong University, Xi’an, Shaanxi 710004, P.R. China; ^3^ Department of Vascular Surgery, The First Affiliated Hospital of Xi’an Jiaotong University, Xi’an, Shaanxi 710061, P.R. China; ^4^ Department of Oncological Surgery, Shaanxi University of Chinese Medicine, Xianyang, Shaanxi 712046, P.R. China; ^5^ Department of Radiation Oncology, The First Affiliated Hospital of Xi’an Jiaotong University, Xi’an, Shaanxi 710061, P.R. China

**Keywords:** breast cancer, MYC, prognosis, clinicopathology, meta-analysis

## Abstract

**Background:**

Proto-oncogene MYC has been indicated to promote progression of many cancers. However, prognostic and clinicopathological significance of MYC in breast cancer need further evaluation.

**Methods:**

We searched EMBASE and PubMed databases to find useful studies. We analyzed relationships between high MYC expression and prognostic data/ clinicopathological features through hazard ratio (HR) and odds ratio (OR). Each statistical test was two-sided.

**Results:**

There were 29 studies (36 cohorts) with 12621 patients enrolled in our study The MYC overexpression was associated with worse DFS/RFS (disease/relapse free survival) in 11 studies (16 cohorts) with 5390 patients, and OS (overall survival) of 7 studies (8 cohorts) with 2672 patients. Subgroup analysis according to ethnicity/technique/data source displayed that MYC overexpression was associated with poor DFS/RFS in FISH, other technique, all data source and Asian/Non-Asian subgroup, and worse OS in all subgroups. In addition, MYC overexpression was related to large tumor size, high histologic grade, lymph node metastasis, negative hormone receptors and positive Ki67 expression.

**Conclusions:**

Our results showed that MYC overexpression was associated with worse prognosis and high risk of breast cancer, especially in patients with negative hormone receptors, which highlighted the potential of MYC as a significant prognostic biomarker of breast cancer.

## INTRODUCTION

Nearly 2 million new breast cancer cases are diagnosed each year from all around the world and account for the first or second leading cause of cancer death in female from developing and developed country respectively [1, 2]. In addition, breast cancer is a heterogeneous disease with a variety of subtypes and molecular markers and displays multiple clinical outcomes and histological characteristics [3]. At present, we use systemic therapies to improve the survival of breast cancer patients, including surgical treatment, chemotherapy, endocrine therapy or immunotherapy [4]. Unfortunately, some effective therapies are hampered by existing biomarkers and the prognosis of breast cancer patients still doesn’t meet our expectations. Thus, searching new biomarkers and therapeutic targets is very significant for patients with invasive breast cancer [5]. New and more effective biomarkers should be explored to predict prognosis and make best therapeutic choice [6].

Proto-oncogene MYC, also named c-Myc and bHLH transcription factor, is an indispensable signal core in a variety of biological processes that support the growth of various types of cancer, such as ovarian cancer, endometrial cancer, breast cancer and so on [7, 8]. MYC regulates the expression of many target genes and non-coding that activate or suppress cell cycle progression, apoptosis, differentiation and control mechanisms of drug resistance [3, 9]. In breast cancer, lots of studies have investigated the significance of MYC. Some studies display positive relationships between MYC overexpression and prognostic/clinicopathological outcome [10-12], while others show contrary results [13-15]. In the past 20 years, there was only one published meta-analysis about MYC and prognostic and clinicopathological significance of breast cancer in 2000 [16]. Though it provided some information, the detection method of MYC expression was very different from that today and the number of included studies with prognosis of breast cancer patients was small. Thus, we need new more systematic studies to acquire high quality and relatively reliable data of prognostic and clinicopathological significance of MYC to stratify breast cancer patients who would benefit from MYC targeted therapy and provide evidence to prospective treatment.

## RESULTS

### Description of included studies

**Figure 1:**
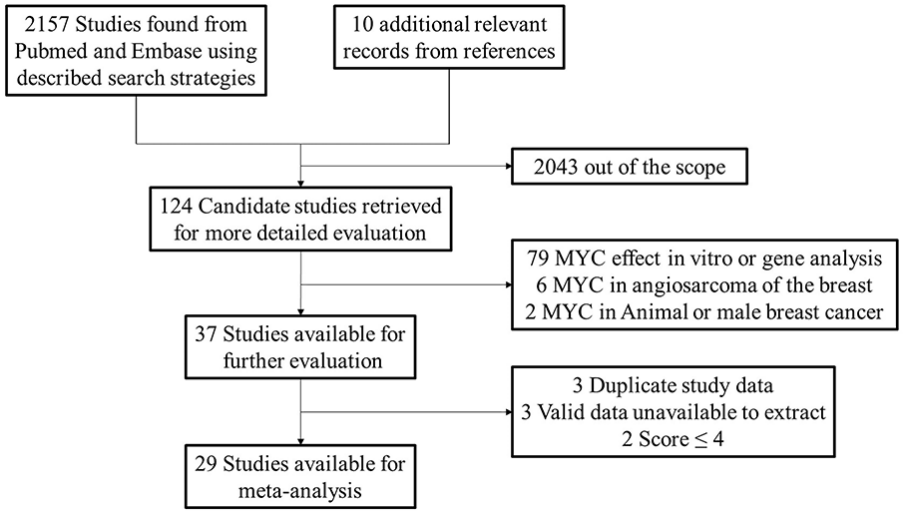
Selection of studies Flow chart showed selection of the studies in the meta-analysis.

We searched 2167 records in total and then selected 124 candidate studies. After further screening, there were 87 studies excluded because of cell experiment, animal specimen, breast angiosarcoma and male patients. Among the remaining studies, three studies [17-19] used the same patient cohorts of other three studies [15, 20, 21] and we chose the high quality studies among them. Then two studies with scores less than 4 and three studies with invalid data were excluded. Ultimately, 29 studies (36 cohorts) were included and the detailed processes of literature search and study selection were shown in Figure 1.

There were 29 studies (36 cohorts) with 12621 breast cancer patients in total involved in our meta-analysis. Among them, 11 studies (16 cohorts) with 5390 patients were available for RFS/DFS survival data and 7 studies (8 cohorts) with 2672 patients were available for OS survival data. 14 (48.3%) studies used FISH method to detect the expression of MYC and the remaining articles applied IHC, qPCR, Genechip, dPCR, SOA and hybridization respectively. All included articles were retrospective. We used the Newcastle-Ottawa quality assessment scale to assess their quality and scores of included studies ranged from 5 to 8 with a mean of 6.966 (Table 1).

**Table 1:** Clinical characteristics of involved studies

First author	Year	Patient source	Type of patients	Technique	Number of corhot	Number of patients	Median/mean age (range) years	Histological grade/stage	No. of patients with MYC overexpression (%)	Follow-up months median (range)	Survival outcome	Scores of study
Sadeghi, S.	2017	Iran	MIX	qPCR	1	104	NA	G1-3	40 (38.46)	NA	NA	5
Gupta, N.	2017	Canada	TNBC	IHC	1	35	54 (30-89)	G1-3	9 (25.71)	30 (0-60)	OS/RFS	7
Green, A. R.	2016	Britain/Canada	MIX	qPCR/IHC	2	1977/1106	NA	G1-3	260 (13.15)/559(50.54)	NA	DSS/DMFS	8
Gogas, H.	2016	Greece	trastuzumab	IHC/ qPCR/FISH	1	119	57 (28–95)	G1-3	10 (8.40)	NA	OS/TTP	8
Mundim, F. G.	2015	Brazil	IDC	IHC	1	80	57 (23-88)	G1-3	69 (86.25)	NA	NA	8
Xu, L. P.	2014	China	IDC, HER2-	IHC	1	166	50 (30-72)	G1-3	46 (27.71)	NA	DSS/DMFS	7
Sengupta, S.	2014	MIX	ERα+	Genechip	2	1129/531	NA	NA	282 (24.98)133(25.05)	NA	RFS	7
Nair, R.	2014	Australia	IDC	FISH	1	272	55 (24-87)	G1-3	46 (16.79)	64 (0-152)	DSS	7
Li, Z.	2014	China	MIX	FISH	1	66	46.3 (23-85)	G1-3	18 (27.27)	NA	NA	5
Li, C.	2014	China	young/old	FISH	2	196/227	(≤35,≥65)	NA	56 (28.57)/30(13.22)	30 (0-60)	OS/DFS	7
He, Y.	2014	China	MIX	IHC	1	168	54.5 (27-82)	G1-3/I-IV	84 (50)	NA	NA	6
Ren, J.	2013	China	MIX	IHC	1	315	NA	I-III	112 (35.56)	49 (13-87)	DFS	7
Pereira, C. B.	2013	Brazil	advanced IDC	IHC/FISH	1	116	52(31-83)	III	36 (31.03)	NA	NA	7
Dueck, A. C.	2013	America	Early-Stage HER2+	IHC	3	584/624/528	50 (22-80)	NA	574 (33)	73.2	DFS	8
Yasojima, H.	2011	Japan	neoadjuvant	FISH	1	100	NA	G1-3/I-III	40 (40.00)	31.6 (3.2-73.0)	RFS	8
Burkhardt, L.	2010	Germany	DCIS	FISH	2	93/92	60.4(34–81)/56.5(28-89)	G1-3	11 (11.82)/6 (6.52)	NA	NA	7
Butt, A. J.	2008	Netherlands\Sweden	MIX	SOA/Genechip	2	295/236	NA	NA	75 (25.42)/47 (19.92)	NA	DFS	8
Rodriguez-P, S. M.	2007	Spain	IBCMFs/IDC	FISH	1	67	NA	G3	25 (37.31)	NA	NA	7
Rodriguez-P, S. M.	2007	Britain	anthracycline	CISH	1	196	NA	G1-3	19 (9.69)	67 (0.5-125)	OS/MFS	7
Linke, S. P.	2006	Switzerland/Germany	Tamoxifen	FISH	1	243	64.3	I-III	28 (11.52)	(0-60)	OS/DSS	8
Aulmann, S.	2006	Germany	locally recurrent	FISH	1	49	50 (26-85)	G1-3	11 (22.44)	23.7 (5 to 63)	OS/RFS	7
Park, K.	2005	South Korea	MIX	FISH	1	208	NA	G1-3	33 (15.87)	51 (18-66)	DFS	6
Al-Kuraya, K.	2005	Saudi Arabia	MIX	FISH	1	152	47 (28-85)	G1-3	24 (15.79)	NA	NA	7
Al-Kuraya, K.	2004	Switzerland	MIX	FISH	1	1504	62 (26-101)	G1-3	79 (5.25)	68 (1–176)	OS	8
Schlotter, C. M.	2003	Germany	node-negative	dPCR	1	181	NA	G1-3	39 (21.5)	42(36-95)	DFS	7
Naidu, R.	2002	Malaysia	MIX	IHC/dPCR	1	399	NA	G1-3	184 (46.12)	NA	NA	6
Rummukainen, J. K.	2001	Finland	MIX	FISH/CISH	1	177	61.6	G1-3	27 (15.25)	81.6 (61.2–93.6)	DMFS	6
Scorilas, A.	1999	Greece	no distant metastasis	hybridization	1	152	60 (24-92)	G1-3	43 (28.29)	60(48-96)	OS/DFS	6
Bieche, I.	1999	France	MIX	qPCR	1	134	58.3(34-91)	G1-3	29 (21.64)	98.4 (12-190.8)	NA	7

MIX: the data is mixed; NA: not available; IDC: invasive ductal carcinoma; DCIS: ductal carcinoma in situ; IBCMFs: invasive breast carcinomas with medullary features; HER-2: human epidermal growth factor 2; IHC: immunohistochemistry; qPCR: real-time quantitative PCR; dPCR: differential PCR; FISH: fluorescence in situ hybridization; CISH: chromogenic in situ hybridisation; SOA: spotted oligonucleotide arrays; TNBC: triple negative breast cancer; TTP: time to progression; OS: over survival; DSS: disease specific survival; DMFS: distant metastasis free survival; MFS: metastasis free survival; RFS/DFS: relapse/disease free survival.

### Data synthesis: clinicopathological features

Our meta-analysis showed that overexpression of MYC significantly correlated to large tumor size, OR=1.269 (1.030-1.563); high histologic grade, OR= 2.151 (1.623-2.851); lymph node metastasis, OR=1.466 (1.115-1.928); negative ER status, OR=1.810 (1.285-2.551); negative PR status, OR=1.545 (1.099-2.173); positive Ki67 expression, OR=2.212 (1.526-3.206). However, high MYC expression wasn’t associated with age, OR=0.865 (0.737-1.015); stage, OR=1.082 (0.683-1.715); HER-2 status, OR=0.571 (0.249-1.312); TNBC phenotype, OR=1.301 (0.590-2.868); Menopausal status, OR=0.882 (0.730-1.066). All of these results were shown in Table 2.

**Table 2:** Meta-analysis for the association of MYC overexpression and clinicopathological features of breast cancer patients

Clinicopathological features	No. of studies	No. of corhot	No. of patients	Model	OR (95% CI)	*P*-value	Heterogeneity		
							*I*2	*I*2 (%)	*P*-value
Age (≥50 vs. <50)	7	7	2932	Fixed	0.865 (0.737-1.015)	0.075	3.73	0.0	0.713
Size (>2cm vs. ≤2cm)	10	11	7118	Random	1.269 (1.030-1.563)	0.025	17.36	42.4	0.067
Histologic grade (G3 vs. G1-2)	16	18	8358	Random	2.151 (1.623-2.851)	0	68.38	75.1	0
lymph node status (N1-3 vs. N0)	14	14	4892	Random	1.466 (1.115-1.928)	0.006	25.57	49.2	0.019
Stage (III-IV vs. I-II)	3	3	413	Fixed	1.082 (0.683-1.715)	0.737	1.88	0.0	0.391
ER status (Negative vs. Positive)	11	12	5953	Random	1.810 (1.285-2.551)	0.001	41.94	73.8	0
PR status (Negative vs. Positive)	10	11	5542	Random	1.545 (1.099-2.173)	0.012	29.16	65.7	0.001
HER-2 status (Negative vs. Positive)	9	10	4153	Random	0.571 (0.249-1.312)	0.187	128.68	93.0	0
TNBC (Yes vs. No)	4	5	3552	Random	1.301 (0.590-2.868)	0.514	41.25	90.3	0
Ki67 status (Positive vs. Negative)	7	7	1918	Random	2.212 (1.526-3.206)	0	12.71	52.8	0.048
Menopausal status (Post vs. Pre)	3	3	1970	Fixed	0.882 (0.730-1.066)	0.194	2.08	3.9	0.353

ER: estrogen receptor; PR: progesterone receptor; HER-2: human epidermal growth factor receptor-2.

### Data synthesis: disease/relapse free survival

**Figure 2:**
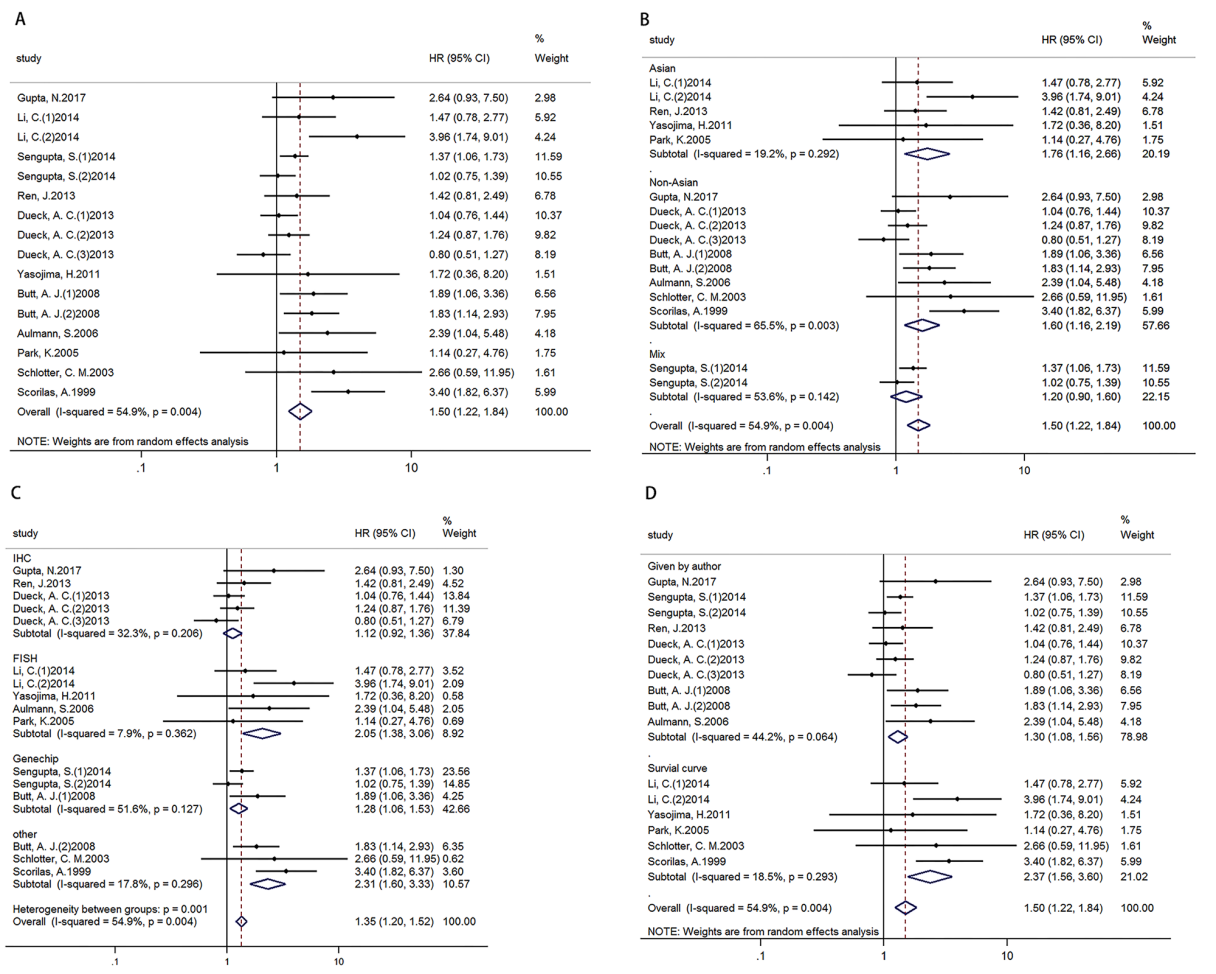
Forest plots of HR for the relationships of MYC overexpression and DFS/RFS Survival data were reported as DFS/RFS **(A)**, as well as subgroup analysis of ethnicity **(B)**, technique **(C)** and data sources **(D)** among included studies.

Analysis of 11 studies (16 cohorts) with 5390 breast cancer patients displayed that high MYC expression was associated with poor DFS/RFS, HR=1.500 (1.224-1.838) (Figure 2A). In addition, results of subgroup analysis according to ethnicity (Figure 2B)/ technique (Figure 2C)/ data sources (Figure 2D) showed that high MYC expression was associated with poor DFS/RFS in Asian and non-Asian subgroups, FISH and other technique subgroups, and two different data source subgroups. (Table 3)

**Table 3:** Main meta-analysis results

Analysis	No. of studies	No. of cohort	No. of patients	Model	HR (95% CI)	P-value	Heterogeneity		
							*I*2	*I*2 (%)	*P*-value
**DFS/RFS**	11	16	5390	Random	1.500 (1.224-1.838)	0	33.29	54.9	0.004
**Ethnicity**									
Asian	4	5	1046	Fixed	1.727 (1.214-2.456)	0.002	4.95	19.2	0.292
Non-Asian	6	9	2684	Random	1.598 (1.164-2.194)	0.004	23.22	65.5	0.003
Mix	1	2	1660	Random	1.201 (0.901-1.601)	0.213	2.15	53.6	0.142
**Technique**									
IHC	3	5	2086	Fixed	1.121 (0.924-1.360)	0.247	5.91	32.3	0.206
FISH	4	5	780	Fixed	2.054 (1.379-3.057)	0	4.34	7.9	0.362
Genechip	2	3	1896	Random	1.300 (0.975-1.732)	0.073	4.13	51.6	0.127
Other	3	3	628	Fixed	2.311 (1.603-3.331)	0	2.43	17.8	0.296
**Data source**									
Given by author	6	10	4326	Random	1.298 (1.080-1.559)	0.005	16.14	44.2	0.064
Survival curve	5	6	1064	Fixed	1.257 (1.108-1.426)	0	6.14	18.5	0.293
**OS**	7	8	2672	Fixed	3.029 (2.385-3.847)	0	9.05	22.7	0.249
**Ethnicity**									
Asian	1	2	423	Fixed	2.795 (1.476-5.293)	0.002	0.67	0	0.414
Non-Asian	6	6	2249	Fixed	3.069 (2.372-3.972)	0	8.31	39.9	0.14
**Technique**									
FISH	3	4	2170	Fixed	2.492 (1.841-3.372)	0	0.93	0	0.817
Other	4	4	502	Fixed	4.191 (2.837-6.190)	0	3.86	22.3	0.249
**Data source**									
Given by author	3	3	397	Fixed	3.586 (2.222-5.787)	0	2.38	16.1	0.304
Survival curve	4	5	2275	Fixed	3.170 (1.760-5.720)	0	6.04	33.7	0.196

### Data synthesis: overall survival

**Figure 3:**
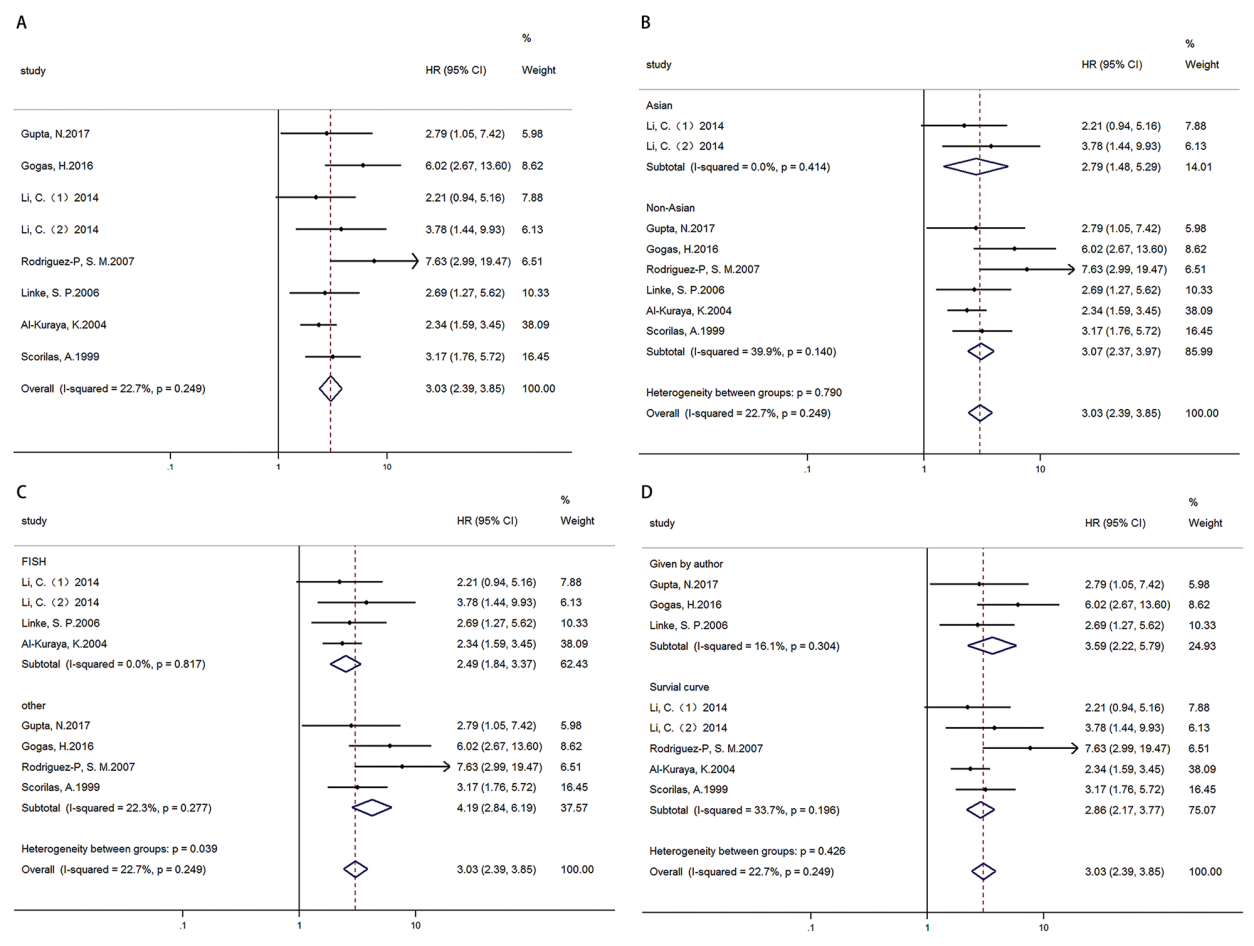
Forest plots of HR for the relationships of MYC overexpression and OS Survival data were reported as OS **(A)**, as well as subgroup analysis of ethnicity **(B)**, technique **(C)** and data sources **(D)** among included studies.

OS was analyzed in 7 articles (8 cohorts) with 2672 patients. Results showed that high MYC expression was associated with poor OS, HR=3.029 (2.385-3.847) (Figure 3A). In addition, results of subgroup analysis by ethnicity (Figure 3B)/ technique (Figure 3C)/ data sources (Figure 3D) showed high MYC expression was associated with poor OS in all ethnicity, technique, data source subgroups respectively (Table 3).

### Publication bias

**Figure 4:**
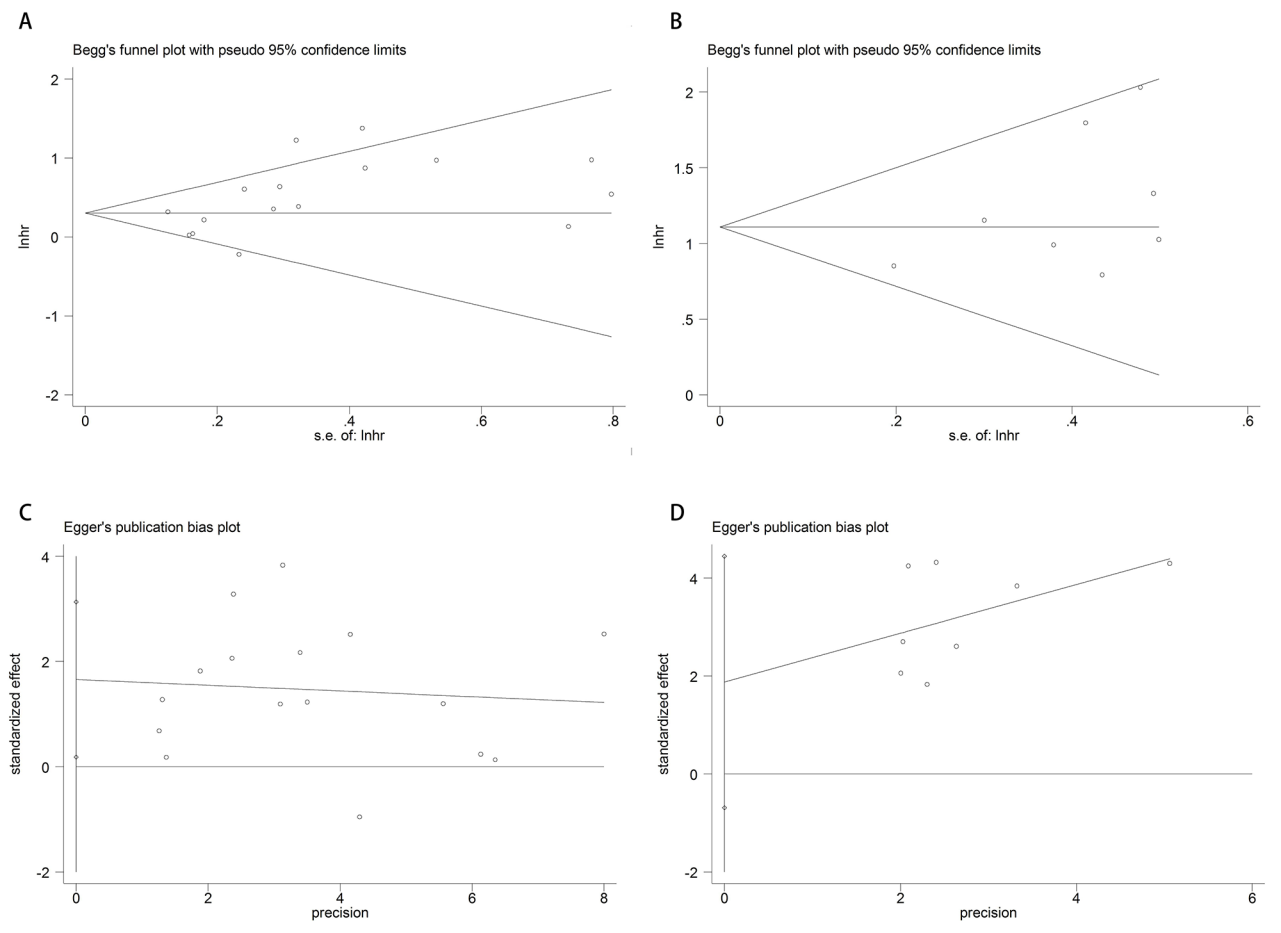
Funnel plots of publication bias of DFS/RFS and OS Begg’s **(A)**/Egger’s **(C)** test of DFS/ RFS and Begg’s **(B)**/Egger’s **(D)** test of OS.

We applied Begg’s /Egger’s test and their funnel plot to assess publication bias. Analysis results of Begg’s /Egger’s test for DFS/RFS and OS were 0.087/ 0.029 (Figure 4A and 4C) and 0.322/0.124 (Figure 4B and 4D) respectively.

### Sensitivity analysis

**Figure 5:**
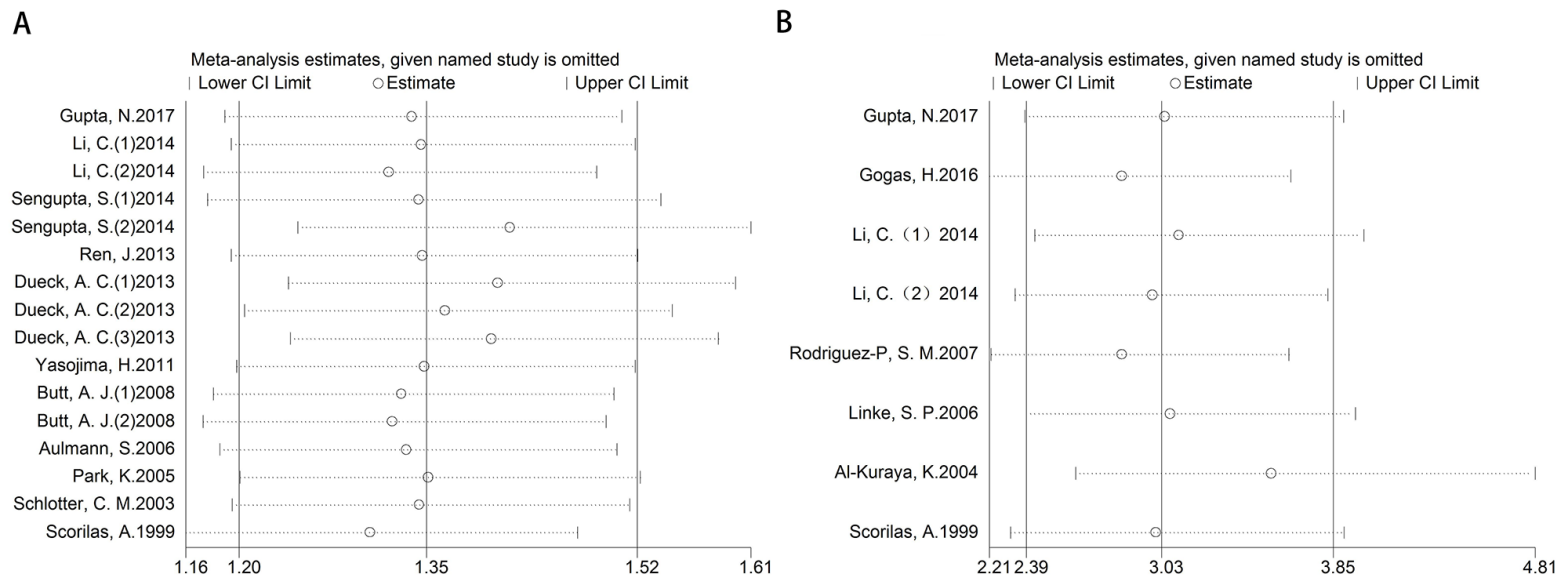
Sensitivity for included studies The effect of single study was evaluated on the whole results of DFS/RFS **(A)** and OS **(B)** in this meta-analysis.

**Figure 6:**
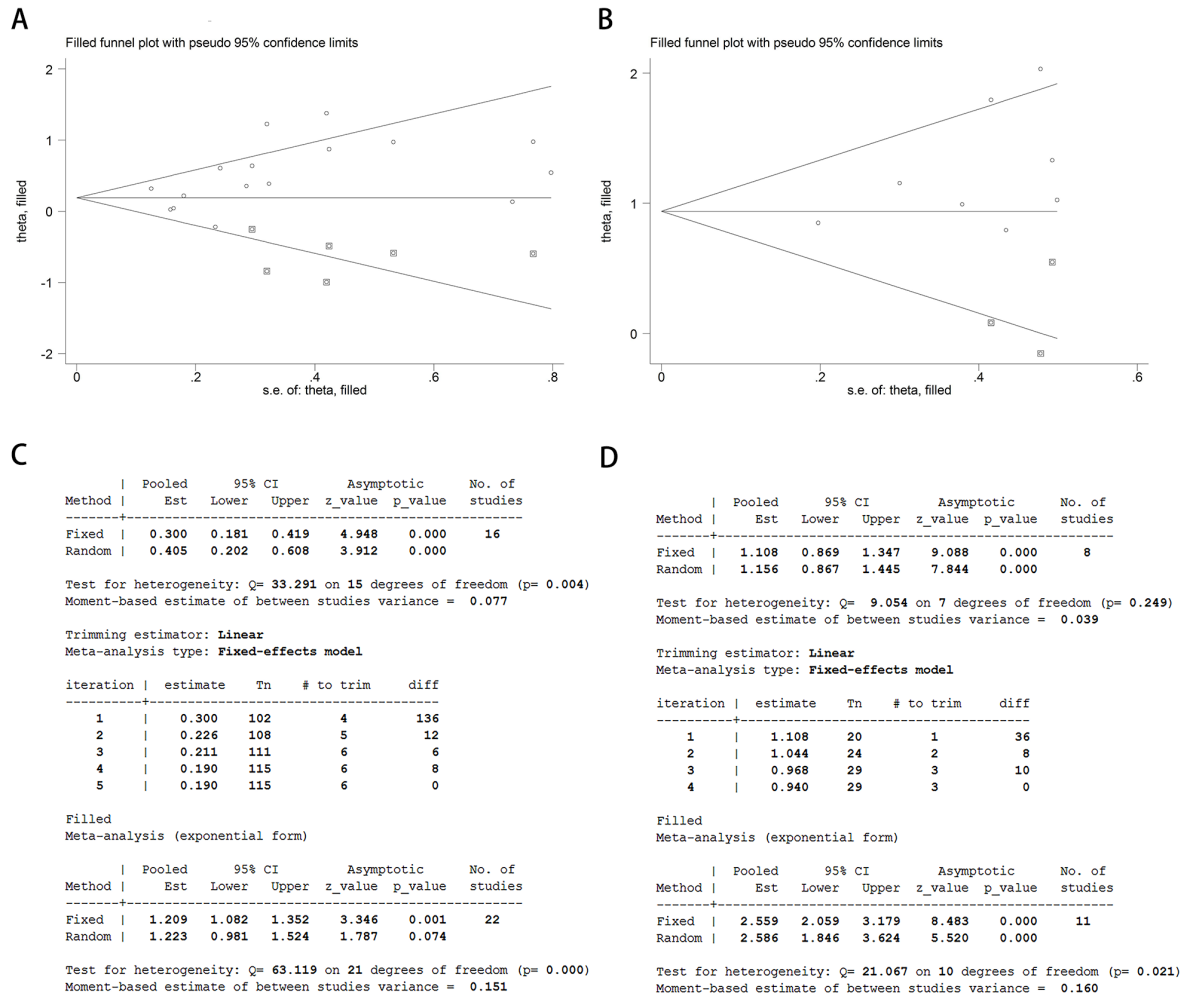
Analysis of trim and fill method for DFS/RFS and OS Funnel plots of trim and fill method for DFS/RFS **(A)** and OS **(B)**. Iterative processes of trim and fill method for DFS/RFS **(C)** and OS **(D)**.

After removing each study at a time, each HR result was shown in Figure 5A-5B. Removal of each study did not change HR significantly both for the DFS/RFS and OS analysis. Furthermore, we used trim and fill method to evaluate the sensitivity of results again. After trimming and filling, the HR tendency of OS did not change (Figure 6B and 6D), however, the HR trend of DFS/RFS was reversed (Figure 6A and 6C).

## DISCUSSION

The proto-oncogene MYC, which encodes a nuclear phosphoprotein transcription factor, plays an important role in various cellular biological processes, such as cell invasion, metabolism, differentiation, proliferation, drug resistance [22]. A lot of clinical researches published before have investigated MYC expression and related signal pathway in breast cancer cells and patients, and discovered strong correlation between MYC overexpression and breast cancer progression [3, 9]. Our results showed that high MYC expression was associated with worse DFS/RFS and OS for breast cancer patients. Besides, MYC overexpression was related to tumor size of more than 2 cm, high histologic grade, lymph node metastasis, negative ER status, negative PR status, positive Ki67 expression. Thus, MYC could be regarded as a potential biomarker and therapeutic target for breast cancer patients.

In our meta-analysis, DFS/RFS displayed moderate heterogeneity. Then subgroup analysis was performed and we found that technique was the origin of heterogeneity. HR of FISH and other technique subgroups in 7 studies (8 cohorts) displayed a poor prognosis of high MYC expression in breast cancer patients, however, the technique of IHC and Genechip (5 studies/ 8 cohorts) showed a negative prognosis of MYC overexpression. These opposite results were mainly because that IHC detected the level of protein, but FISH detected the level of DNA. With regard to subgroup of Genechip, one study (two cohorts) used 2 different cohorts of endocrine therapy but not chemotherapy treated patients and chemotherapy treated patients [23]. This would lead to heterogeneity and got different results. The other subgroups of DFS/RFS, ethnicity and Data source, displayed the same significance of HR excepting for Mix of ethnicity. The reason may be the same as that in Genechip subgroup. The results of OS displayed mild heterogeneity. Though all subgroups of OS showed a positive significance between poor prognosis and high MYC overexpression, further subgroup analysis of OS showed the heterogeneity was also conducted from different technique, the reasons of heterogeneity in technique subgroup were explained as what we discussed above.

Besides, Begg’s/Egger’s test showed there was no evidence of publication bias for OS in regard to high MYC expression, however, Egger’s test displayed, Begg’s test not, some evidence of publication bias in DFS/RFS group. Though both HR results of DFS/RFS and OS showed there was significant between high MYC expression and DFS/RFS/ OS, further analysis of trim and fill method in DFS/RFS showed a reversed result. It indicated that future new studies about this would change in HR result of DFS/RFS. This might be mainly because that the heterogeneity of different technique resulted in this.

Some articles studied the relationships between MYC amplification/overexpression and hormone receptors [17, 24] and found that MYC amplification/overexpression was more frequent in breast cancer without ER or PR expression, that could be used as a potential target in breast cancer of negative hormone receptors. Our meta-analysis also displayed that high MYC expression related to the negative ER and PR. Interestingly, there was no statistical significance of high MYC expression in TNBC and HER-2 status groups, that further showed MYC overexpression could be a target for breast cancer of negative hormone receptors. But because of the limited number of studies, we need more researches to investigate the relationships between MYC overexpression and TNBC phenotype.

Our meta-analysis has significant guided values in breast cancer. Firstly, it indicates that MYC overexpression is associated with poor DFS/RFS and OS, that demonstrates that MYC may be a potential therapeutic target of breast cancer, especially in phenotype of negative hormone receptors. Secondly, MYC referred to invasive biological behavior, including larger tumor size, high histologic grade, lymph node metastasis, positive Ki67 status. If we could combine MYC inhibitor and chemotherapy in the future, it should dramatically increase survival time of patients suffered from invasive breast cancer. Unfortunately, we are short of pharmacological efficacy of direct MYC inhibitors at present [25], many scientists have shifted their directions on active MYC signal pathways and further investigating the target genes.

Of course, there are still limitations in our meta-analysis. In the first place, identifications of high MYC expression in included studies aren’t exactly the same and different techniques might be the source of heterogeneity and lead to contrary results. Besides, Egger’s test of DFS/RFS showed there was statistical significance and further analysis of trim and fill method in DFS/RFS displayed a reversed result. It means, in the future new studies might change our DFS/RFS results of meta-analysis. Although Begg’s and Egger’s test of OS showed that there was no statistical significance. We should cautiously understand these results, because just available HR or K-M survival curves were included, and technique was still the source of heterogeneity in OS.

In short, this meta-analysis implies that high MYC expression in breast cancer is related to poor prognosis of patients, especially to patients with negative ER and PR. And more studies about the relationships between DFS/RFS and MYC over expression need be done in the future, different techniques of detecting MYC might lead to discrepancy results. Combination therapy of MYC signal pathway inhibitors would improve clinical outcomes of breast cancer patients, especially for patients with negative hormone receptors.

## MATERIALS AND METHODS

### Literature search

Our meta-analysis was processed according to PRISMA guidelines. Studies were extracted by searching PubMed and EMBASE databases commencing 1997 through July, 2017 by using the search words “MRTL OR MYCC OR c-Myc OR bHLHe39 OR MYC AND breast cancer”. We firstly scanned titles and abstracts to exclude unrelated and review studies. Then we made finally decision to choose useful studies by reading the full text. Associated references from included studies were manually searched to add relevant articles.

### Inclusion and exclusion

All of our included studies satisfied the following inclusion criteria: 1) diagnosis of breast cancer was proven by pathologists; 2) investigating the relationships between high MYC expression and DFS/RFS, OS, or clinicopathological data in breast cancer patients; 3) provided the data of HR and 95% CIs, or Kaplan-Meier survival curves of DFS/RFS or OS, which provided us available data to extract HR and 95% CI. 4) NOS score ≥ 5. Exclusion criteria: 1) no available data of prognostic or clinicopathological information and the data could not be applied to calculate from Kaplan-Meier survival curve; 2) NOS score ≤ 4.

### Data extraction

Two reviewers (Jingkun Qu and Xixi zhao) searched and evaluated the studies independently. The following information was extracted from every included study, including first author name, published year, breast cancer patients source, type of patients, age, patients number, detecting technique, high MYC expression (%), follow-up time, DFS/RFS/OS and other clinicopathological features. If the univariate and multivariate analysis were both available, the multivariate results were chosen. If the above information was not found, we used “NA (not available)” to mark.

### Quality of the studies

We applied the Newcastle-Ottawa Scale to evaluate the quality of every included study [26].

### Statistical analysis

HR and 95% CIs were applied to investigate the relationships between high MYC expression and DFS/RFS/OS. If survival information was only available in the form of figures, we scanned Kaplan-Meier survival curves through Engauge Digitizer version 4.1 (free Engauge Digitizer could be acquired on http://sourceforge.net) and recovered survival information of HR and 95%CI [27, 28]. Information of clinicopathology was extracted in available studies to calculate OR by Stata. The analysis of heterogeneity, publication bias and sensitivity were describe as before [6]. Statistical analysis was processed by Stata 14.0 (Stata Corporation, College Station, TX).

## References

[1] Siegel RL, Miller KD, Jemal A (2017). Cancer statistics, 2017. CA Cancer J Clin.

[2] Torre LA, Bray F, Siegel RL, Ferlay J, Lortet-Tieulent J, Jemal A (2015). Global cancer statistics, 2012. CA Cancer J Clin.

[3] Fallah Y, Brundage J, Allegakoen P, Shajahan-Haq AN (2017). MYC-driven pathways in breast cancer subtypes. Biomolecules.

[4] Matsen CB, Neumayer LA (2013). Breast cancer: a review for the general surgeon. JAMA Surg.

[5] Petekkaya I, Ayyildiz V, Kizilarslanoglu MC, Sahin U, Gezgen G, Roach EC, Karcaaltincaba M, Altundag K (2012). Prognosis of breast cancer in patients with peritoneal metastasis. Breast.

[6] Zhao X, Qu J, Sun Y, Wang J, Liu X, Wang F, Zhang H, Wang W, Ma X, Gao X, Zhang S (2017). Prognostic significance of tumor-associated macrophages in breast cancer: a meta-analysis of the literature. Oncotarget.

[7] Cichon MA, Moruzzi ME, Shqau TA, Miller E, Mehner C, Ethier SP, Copland JA, Radisky ES, Radisky DC (2016). MYC is a crucial mediator of TGFbeta-induced invasion in basal breast cancer. Cancer Res.

[8] Cancer Genome Atlas Research N (2011). Integrated genomic analyses of ovarian carcinoma. Nature.

[9] Kalkat M, De Melo J, Hickman KA, Lourenco C, Redel C, Resetca D, Tamachi A, Tu WB, Penn LZ (2017). MYC deregulation in primary human cancers. Genes (Basel).

[10] Butt AJ, Sergio CM, Inman CK, Anderson LR, McNeil CM, Russell AJ, Nousch M, Preiss T, Biankin AV, Sutherland RL, Musgrove EA (2008). The estrogen and c-Myc target gene HSPC111 is over-expressed in breast cancer and associated with poor patient outcome. Breast Cancer Res.

[11] Aulmann S, Adler N, Rom J, Helmchen B, Schirmacher P, Sinn HP (2006). c-myc amplifications in primary breast carcinomas and their local recurrences. J Clin Pathol.

[12] Scorilas A, Trangas T, Yotis J, Pateras C, Talieri M (1999). Determination of c-myc amplification and overexpression in breast cancer patients: evaluation of its prognostic value against c-erbB-2, cathepsin-D and clinicopathological characteristics using univariate and multivariate analysis. Br J Cancer.

[13] Ren J, Jin F, Yu Z, Zhao L, Wang L, Bai X, Zhao H, Yao W, Mi X, Wang E, Olopade OI, Wei M (2013). MYC overexpression and poor prognosis in sporadic breast cancer with BRCA1 deficiency. Tumour Biol.

[14] Knudsen ES, McClendon AK, Franco J, Ertel A, Fortina P, Witkiewicz AK (2015). RB loss contributes to aggressive tumor phenotypes in MYC-driven triple negative breast cancer. Cell Cycle.

[15] Dueck AC, Reinholz MM, Geiger XJ, Tenner K, Ballman K, Jenkins RB, Riehle D, Chen B, McCullough AE, Davidson NE, Martino S, Sledge GW, Kaufman PA (2013). Impact of c-MYC protein expression on outcome of patients with early-stage HER2+ breast cancer treated with adjuvant trastuzumab NCCTG (alliance) N9831. Clin Cancer Res.

[16] Deming SL, Nass SJ, Dickson RB, Trock BJ (2000). C-myc amplification in breast cancer: a meta-analysis of its occurrence and prognostic relevance. Br J Cancer.

[17] Perez EA, Jenkins RB, Dueck AC, Wiktor AE, Bedroske PP, Anderson SK, Ketterling RP, Sukov WR, Kanehira K, Chen B, Geiger XJ, Andorfer CA, McCullough AE (2011). C-MYC alterations and association with patient outcome in early-stage HER2-positive breast cancer from the north central cancer treatment group N9831 adjuvant trastuzumab trial. J Clin Oncol.

[18] Choschzick M, Lassen P, Lebeau A, Marx AH, Terracciano L, Heilenkotter U, Jaenicke F, Bokemeyer C, Izbicki J, Sauter G, Simon R (2010). Amplification of 8q21 in breast cancer is independent of MYC and associated with poor patient outcome. Mod Pathol.

[19] Tan DS, Marchio C, Jones RL, Savage K, Smith IE, Dowsett M, Reis-Filho JS (2008). Triple negative breast cancer: molecular profiling and prognostic impact in adjuvant anthracycline-treated patients. Breast Cancer Res Treat.

[20] Rodriguez-Pinilla SM, Jones RL, Lambros MB, Arriola E, Savage K, James M, Pinder SE, Reis-Filho JS (2007). MYC amplification in breast cancer: a chromogenic in situ hybridisation study. J Clin Pathol.

[21] Al-Kuraya K, Schraml P, Torhorst J, Tapia C, Zaharieva B, Novotny H, Spichtin H, Maurer R, Mirlacher M, Kochli O, Zuber M, Dieterich H, Mross F (2004). Prognostic relevance of gene amplifications and coamplifications in breast cancer. Cancer Res.

[22] Singhi AD, Cimino-Mathews A, Jenkins RB, Lan F, Fink SR, Nassar H, Vang R, Fetting JH, Hicks J, Sukumar S, De Marzo AM, Argani P (2012). MYC gene amplification is often acquired in lethal distant breast cancer metastases of unamplified primary tumors. Mod Pathol.

[23] Sengupta S, Biarnes MC, Jordan VC (2014). Cyclin dependent kinase-9 mediated transcriptional de-regulation of cMYC as a critical determinant of endocrine-therapy resistance in breast cancers. Breast Cancer Res Treat.

[24] Pereira CB, Leal MF, de Souza CR, Montenegro RC, Rey JA, Carvalho AA, Assumpcao PP, Khayat AS, Pinto GR, Demachki S, de Arruda Cardoso Smith M, Burbano RR (2013). Prognostic and predictive significance of MYC and KRAS alterations in breast cancer from women treated with neoadjuvant chemotherapy. PLoS One.

[25] Hartl M (2016). The quest for targets executing MYC-dependent cell transformation. Front Oncol.

[26] Zhao Y, Dai C, Wang M, Kang H, Lin S, Yang P, Liu X, Liu K, Xu P, Zheng Y, Li S, Dai Z (2016). Clinicopathological and prognostic significance of metastasis-associated in colon cancer-1 (MACC1) overexpression in colorectal cancer: a meta-analysis. Oncotarget.

[27] Zhang QW, Liu L, Gong CY, Shi HS, Zeng YH, Wang XZ, Zhao YW, Wei YQ (2012). Prognostic significance of tumor-associated macrophages in solid tumor: a meta-analysis of the literature. PLoS One.

[28] Zhao X, Qu J, Hui Y, Zhang H, Sun Y, Liu X, Zhao X, Zhao Z, Yang Q, Wang F, Zhang S (2017). Clinicopathological and prognostic significance of c-Met overexpression in breast cancer. Oncotarget.

